# Flavonoids are identified from the extract of Scutellariae Radix to suppress inflammatory-induced angiogenic responses in cultured RAW 264.7 macrophages

**DOI:** 10.1038/s41598-018-35817-2

**Published:** 2018-11-27

**Authors:** Guowei Gong, Huaiyou Wang, Xiangpeng Kong, Ran Duan, Tina T. X. Dong, Karl W. K. Tsim

**Affiliations:** 1 Department of Bioengineering, Zunyi Medical University, Zhuhai Campus, Zhuhai, Guangdong, 519041 China; 2 Shenzhen Key Laboratory of Edible and Medicinal Bioresources, The Hong Kong University of Science and Technology, Shenzhen, 518057 China; 3 Division of Life Science, Center for Chinese Medicine, The Hong Kong University of Science and Technology, Clear Water Bay, Hong Kong China

## Abstract

Scutellariae Radix (SR), also named Huangqin in China, is the dried root of *Scutellaria baicalensis* Georgi. Historically, the usage of SR was targeted to against inflammation. In fact, chronic inflammation has a close relationship with hypoxia and abnormal angiogenesis in tumor cells. Hence, we would like to probe the water extract of SR in suppressing the inflammation-induced angiogenesis. Prior to determine the pharmaceutical values of SR, the first step is to analysis the chemical compositions of SR according to China Pharmacopeia (2015). From the results, the amount of baicalin was 12.6% by weight. Furthermore, the anti-angiogenic properties of SR water extract were evaluated in lipopolysaccharide (LPS) pre-treated cultured macrophage RAW 264.7 cells by detecting the inflammatory markers, i.e. Cox-2, cytokine and iNOS, as well as the translocation activity of NFκB and angiogenic biomarker, i.e. VEGF. This herbal extract was capable of declining both inflammatory and angiogenic hallmarks in a concentration-dependent manner. Moreover, the SR-derived flavonoids, i.e. baicalin, baicalein, wogonin and wogonoside, were shown to be active chemicals in the anti-inflammatory-induced angiogenesis. Therefore, the inflammation-induced angiogenesis is believed to be suppressed by SR water extract, or its major ingredients. These results shed light in the benefiting role of SR in the inflammation-induced angiogenesis *in vitro*.

## Introduction

Inflammation is triggered by harmful stimuli, such as pathogens, damaged cells or irritants. Indeed, this immune response involves immune cells, blood vessels and other types of mediators^1^. Removing of the injured tissue and initiating body repair are the major responsibilities of inflammation^2^. Nevertheless, the hyper-activated inflammation could be rather problematic: because the over reactive inflammatory response can provoke allergies and auto-immune diseases, e.g. arthritis. In addition, the highly activated inflammation is able to trigger skin damage and pain^3,4^. Moreover, chronic inflammation has a close relationship with cancer development^5^. In tumor’s micro-environment, a high content of inflammatory cells has been found, and these cells are believed to participate in neoplastic process, fostering proliferation, survival and migration of cancer cells^6^. Therefore, a proper regulation of inflammation is believed to post a great challenging for human health.

Traditional Chinese medicines (TCMs) have long history of usage as health food supplement and/or medicine to treat diseases in China. Scutellariae Radix (SR; the root *Scutellaria baicalensis* Georgi.), also named as Huangqin in China, was first recorded in Shennong Bencao Jing in AD 200 to 250. Indeed, SR is a classical nutraceutical herb being described in a large number of Chinese medicine prescriptions^7^. Many herbal formulae contain SR, and the major functions of these herbal mixtures are to mitigate inflammation. One of these commonly used recipes containing SR as the major herb is Huang Qin Tang, recorded by Zhang Zhongjing (AD ~984) in Shanghan Lun. According to TCMs theory, the role of this decoction is to clear heat dampness and purge fire^8^. Therefore, SR is considered as an indispensable herb in Chinese literature to eliminate heat/fire, i.e. detoxification and anti-inflammation.

Angiogenesis is a critical constituent of inflammation, and, classically, tumor angiogenesis is also interpreted as an inflammation-induced angiogenesis^9^. The tumor tissue, exhibiting an excessively active process of angiogenesis, is composed of predominant inflammatory infiltrate, neoplastic and stromal cells^9^. Indeed, vascular endothelial growth factor (VEGF) is a critical player in modulating angiogenesis development, and which is believed to be secreted by immune cells^10^. The innate immune cell, particularly macrophage, is reported to express several VEGF receptors (VEGFRs)^11,12^. Macrophages are being recruited in responding to the receptor stimulation, and which significantly contribute to the process of angiogenesis^13^. Furthermore, it is estimated that approximately 15–20% of malignancies are triggered by chronic inflammation^9^. The initiation and progression of cancer are also closely linked to angiogenesis. Here, we would like to probe the possible anti-angiogenic functions of SR in cultured macrophage RAW 264.7 cells. In addition, the chemicals deriving from SR exctract responsible for this function was identified. The angiogenic biomarkers, e.g. Cox-2, cytokines, iNOS, VEGF, were determined *in vitro* by the challenging of SR herbal extract and/or its active ingredients.

## Results

### SR suppresses inflammation

**Figure 1:**
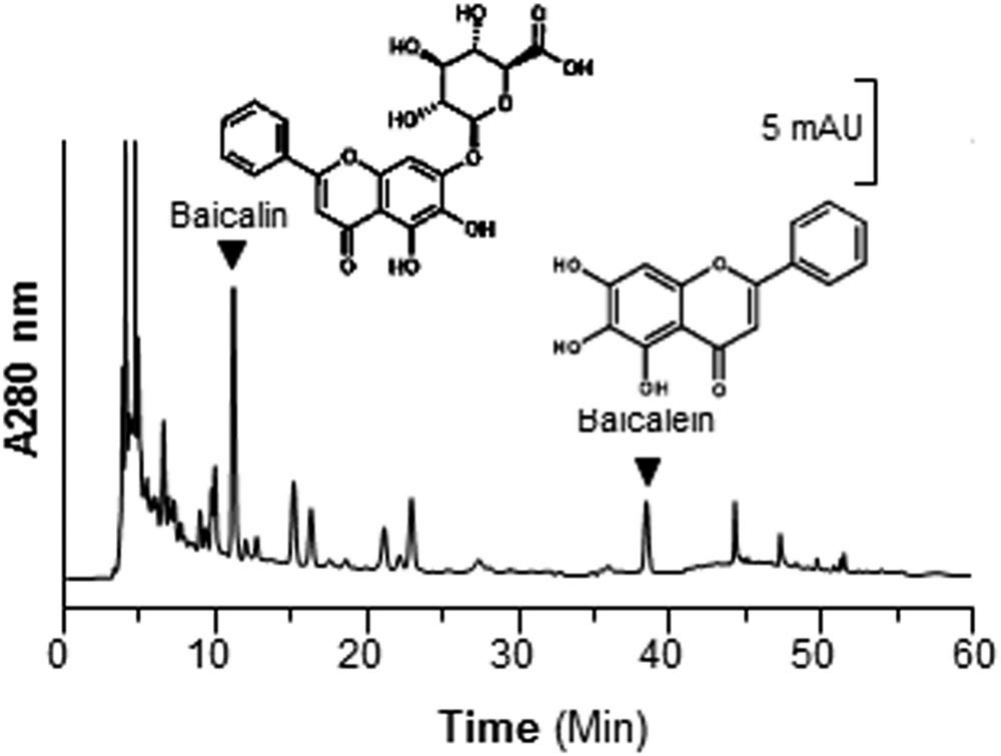
Fingerprint of water extract of SR. Ten μL of 100 mg/mL of SR water extract was subjected to HPLC-DAD analysis, and the chemical fingerprint was revealed at the wavelength 280 nm. The identification and chemical structures of baicalin and baicalein were shown here. Representative chromatograms were shown, *n* = 3.

Chemical standardization of SR water extract was required to ensure the repeatability of the below biochemical assays. According to Chinese Pharmacopeia (2015) requirement, the content of baicalin in SR extract should be higher than 9.0%. From the HPLC results, the baicalin content of the prepared SR water extract was 12.6%, which was much higher than the minimum requirement. In addition, the amount of baicalein was higher than 10% of the dry material, as determined by chemical analysis. Indeed, these two chemicals are the major flavonoids in SR. Besides, the HPLC fingerprint of SR water was achieved at an absorbance of 280 nm **(**Fig. 1**)**.

**Figure 2:**
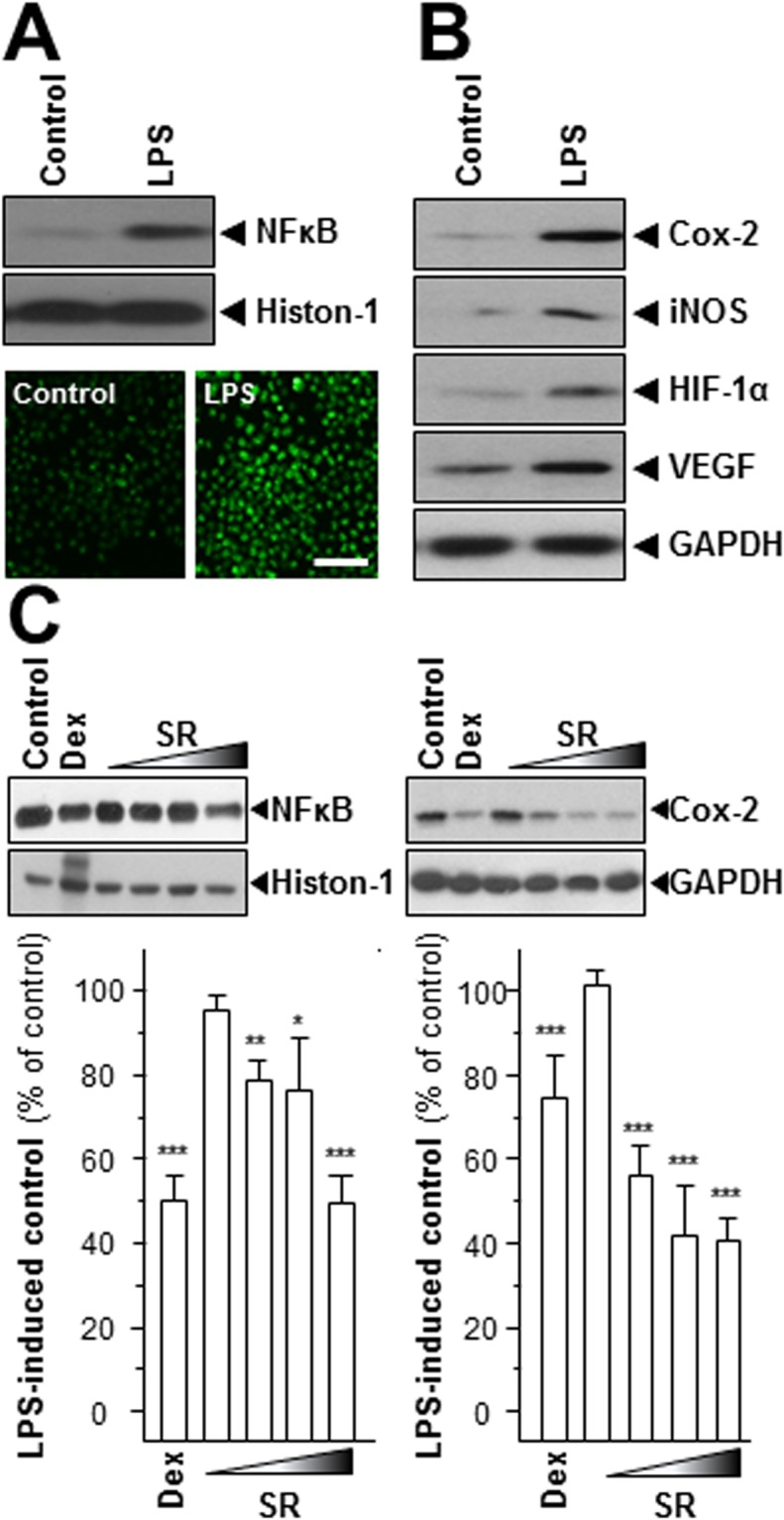
SR suppresses the expressions of NFκB and Cox-2. LPS (1 μg/mL) were utilized for 24 hours as mimicking inflammatory condition. (**A**,**B**) The translational levels of Cox-2 (~72 kDa), iNOS (~130 kDa), HIF-1α (~90 kDa) and VEGF (~27 kDa) and nuclear protein of NFκB (~60 kDa) were detected by immunoblot analysis. Histon-1 (~27 kDa) acted as nuclear internal control and GAPDH (~38 kDa) served as an cytosolic internal control. Cells were labeled with fluorescent NO indicator DAF-FM DA for 30 min. The amounts of NO were evaluated by measuring the fluorescence intensity. Micrographs were taken by a confocal microscope (lower panel), Bar = 100 µm. (**C**) Cells were treated with various concentrations of SR extracts (0.03, 0.1, 0.3, 1.0 mg/mL) for 48 hours. The nuclear protein of NFκB was isolated and detected by immunoblot analysis using specific antibodies. The translational level of Cox-2 was detected by specific antibodies (upper panel), and GAPDH served as an internal control. Here, dexamethasone (Dex; 10 μM) served as positive control. All data were exhibited as the percentage of LPS-induced maximum blank reading (lower panel), in Mean ± SEM, where *n* = 3. Statistically significant changes were classified as significant (*) where *p* < 0.05 more significant (**) where *p* < 0.01 and highly significant (***) where *p* < 0.001.

Application of lipopolysaccharide (LPS) on cultured RAW 264.7 cells was a well-studied model to mimic inflammatory condition^14^. In the LPS-treated cells, the transcription factor NFκB was induced to translocate from cytosol into nucleus robustly, as illustrated here by both western blotting data of nucleus-isolated fraction and immuno-histochemical staining (Fig. 2A). In addition, the expressions of angiogenic biomarkers, including Cox-2, iNOS, HIF-1α and VEGF, were markedly induced by LPS stimulation **(**Fig. 2B**)**. These proteins are closely related to the inflammation-induced angiogenesis in cultured macrophage. In LPS-applied RAW 264.7 cells, the amount of NFκB in nucleus fraction was reduced strikingly and the deduction was linear **(**Fig. 2C**)**. The maximum inhibition was at ~50%, as compared to the blank **(**Fig. 2C**)**. Cox-2 is a mediator for angiogenesis and tumor growth^15^ and NFκB is able to regulate Cox-2 expression in various types of cancer cells^16,17^. Once NFκB being activated, i.e. during the inflammatory situation, it could be translocated into nucleus as to regulate transcription of Cox-2 gene. The expression level of Cox-2 was shown in similar pattern with that of NFκB, and the inhibitory effect was in a dose-dependent manner **(**Fig. 2C**)**. Dexamethasone served as a positive control, a well-studied synthesized drug to mitigate inflammation clinically, which could suppress NFκB translocation and Cox-2 expression, significantly **(**Fig. 2C**)**.

**Figure 3:**
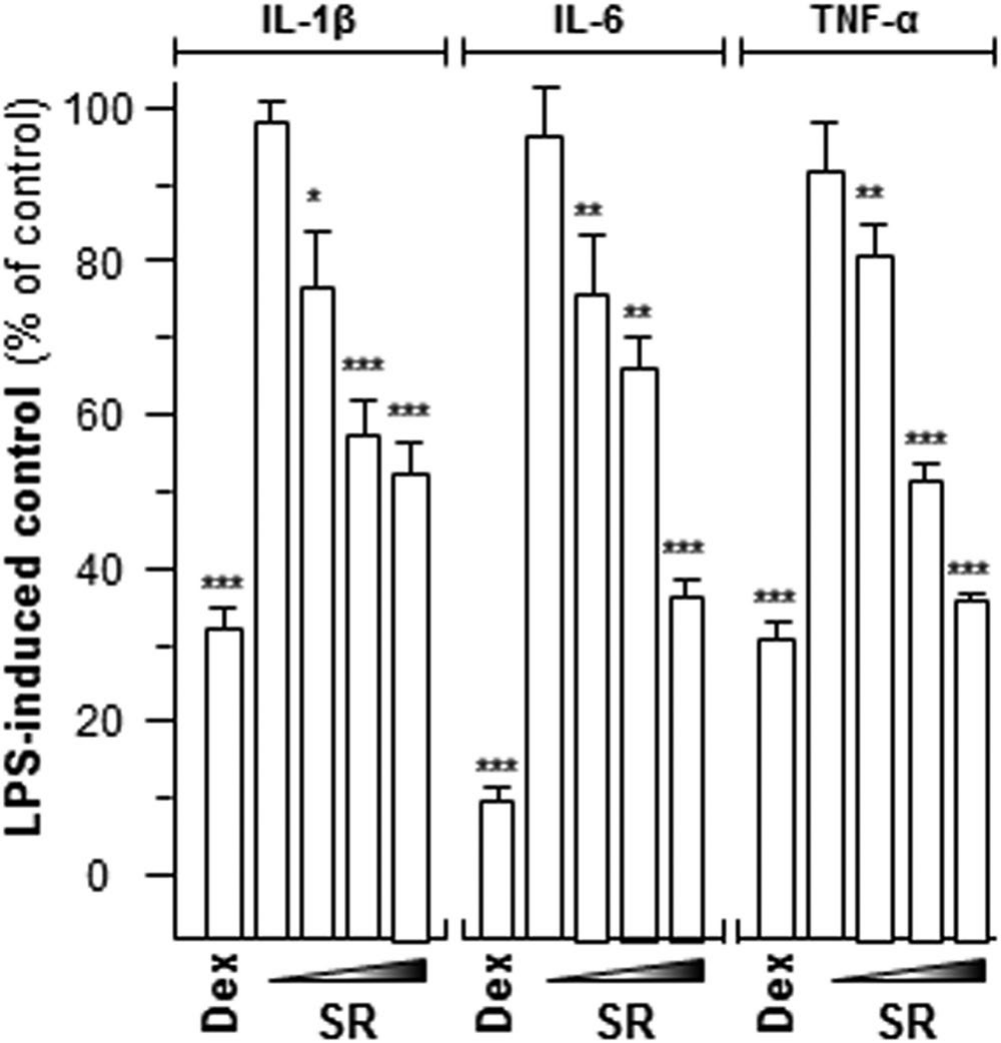
SR modulates cytokine mRNA levels. LPS-stimulated cells were treated with various concentrations of SR extracts (0.03, 0.1, 0.3, 1.0 mg/mL) for 48 hours. Total RNAs were isolated and reverse transcribed to cDNA for PCR analysis. The mRNA levels were determined by the Ct-value method and normalized by the house keeping gene GAPDH rRNA. Here, dexamethasone (Dex; 10 μM) served as positive control. Values were in the percentage of LPS-induced maximum reading, in Mean ± SEM, where *n* = 3. Statistically significant changes were classified as significant (*) where *p* < 0.05 more significant (**) where *p* < 0.01 and highly significant (***) where *p* < 0.001 as compared with control group.

The pro-inflammatory cytokines, i.e. tumor necrosis factor-α (TNF-α), interleukin-1β (IL-1β), and IL-6, activate inflammation, as proposed for the deterioration of angiogenesis in tumor cells^18^. The mRNAs encoding IL-1β, IL-6 and TNF-α were restrained upon the SR treatment **(**Fig. 3**)**. The SR herbal extract (1 mg/mL) showed the strongest inhibition, i.e.~50% for IL-1β, ~60% for IL-6 and ~60% for TNF-α **(**Fig. 3**)**. Dexamethasone was a positive control suppressing cytokine expression at least 70% **(**Fig. 3).

**Figure 4:**
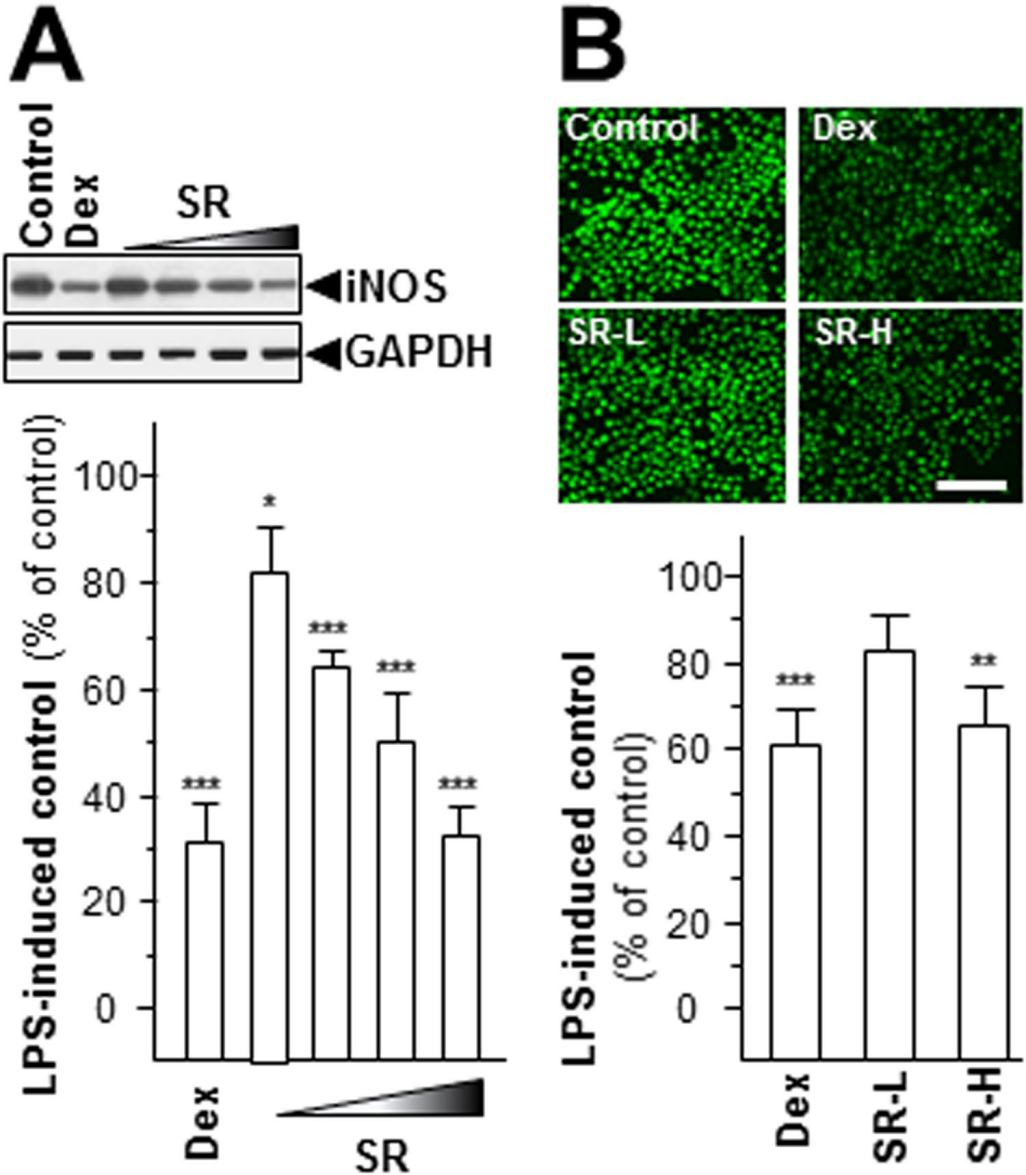
SR declines iNOS and NO productions. (**A**) Various concentrations of SR extracts (0.03, 0.1, 0.3, 1.0 mg/mL) were applied onto LPS-stimulated RAW 264.7 for 48 hours, and cytosolic protein of iNOS (~130 kDa) was detected. GAPDH (~38 kDa) served as an internal control. (**B**) The LPS-stimulated cells were treated with various concentrations of SR (SR-L at 0.03 mg/mL and SR-H at 1.0 mg/mL) for 48 hours, and then labeled with fluorescent NO indicator DAF-FM DA for 30 min. The amounts of NO were evaluated by measuring the fluorescence intensity. Micrographs were taken by a confocal microscope (upper panel), Bar = 100 µm. Here, dexamethasone (Dex; 10 μM) served as positive control. Values were at the percentage of LPS-induced maximum reading (lower panel), in Mean ± SEM, where *n* = 3. Statistically significant changes were classified as significant (*) where *p* < 0.05 more significant (**) where *p* < 0.01 and highly significant (***) where *p* < 0.001 as compared with control group.

One of the main inflammatory mediators reported to be committed in inflammation and carcinogenesis is nitric oxide (NO). There are three regimens for NO synthesis and production, i.e. neuronal (nNOS), endothelial (eNOS), and inducible (iNOS) synthases^19^. Only iNOS is a characteristic for the pathophysiology of inflammation^19^. Here, we revealed the protein level of iNOS in LPS-stimulated RAW 264.7 cells. The expression of LPS-induced iNOS was decreased under application of various concentrations of SR herbal extract, as compared to the control **(**Fig. 4A**)**. The minimal inhibition of iNOS expression was revealed at 0.03 mg/mL SR extract by ~20%. The maximal suppression was at 70% in the present of 1 mg/mL SR extract. Another method was also employed here in detecting NO production: DAF-FM DA was used for the quantification of NO. In cultured RAW 264.7 cells, LPS provoked a progressive rise in intracellular NO production, as reflected by fluorescence intensity **(**Fig. 4 B). The cancellation effects were also observed under the challenging of various contents of SR extract **(**Fig. 4B**)**. Dexamethasone restrained the NO production robustly after 48 hours of treatment **(**Fig. 4B**)**. In both cases, the amounts of iNOS and NO were significantly suppressed in LPS-stimulated RAW 264.7 cells.

### SR restrains expression of tumor angiogenesis marker

**Figure 5:**
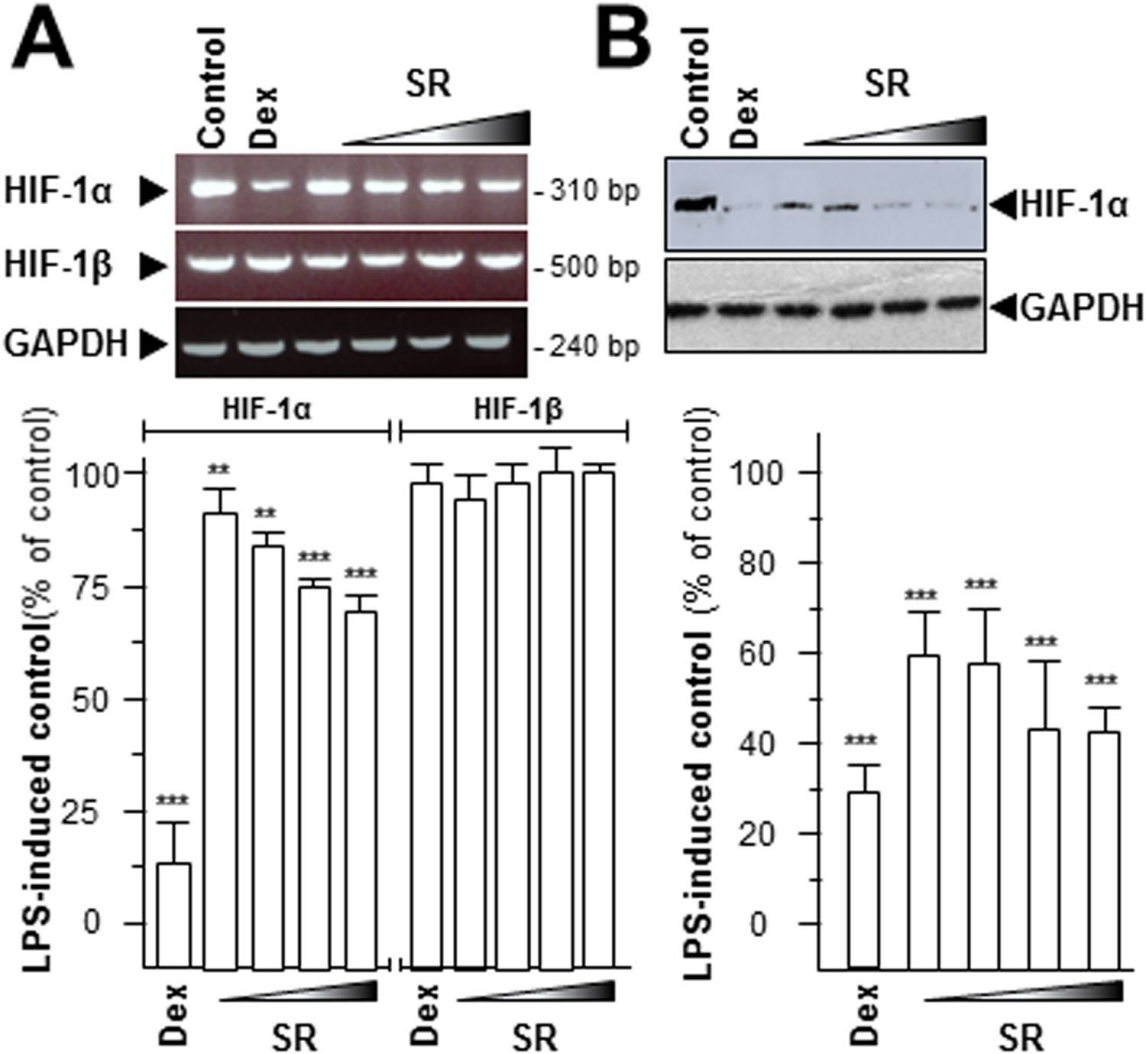
SR decrease HIF-1α mRNA and protein levels. Series dilutions of SR extracts (0.03, 0.1, 0.3, 1.0 mg/mL) were used onto LPS-stimulated RAW 264.7 cells for 48 hours. (**A**) Total RNAs were isolated and reverse transcribed to cDNA for PCR analysis and normalized by the house keeping gene GAPDH. (**B**) The protein level of HIF-1α (~90 kDa) was detected by immunoblot analysis, and GAPDH (~38 kDa) served as an internal control. Here, dexamethasone (Dex; 10 μM) served as positive control. Values were at the percentage of LPS-induced maximum blank reading, in Mean ± SEM, where *n* = 3. Statistically significant changes were classified as significant more significant (**) where *p* < 0.01 and highly significant (***) where *p* < 0.001 as compared with control group.

**Figure 6:**
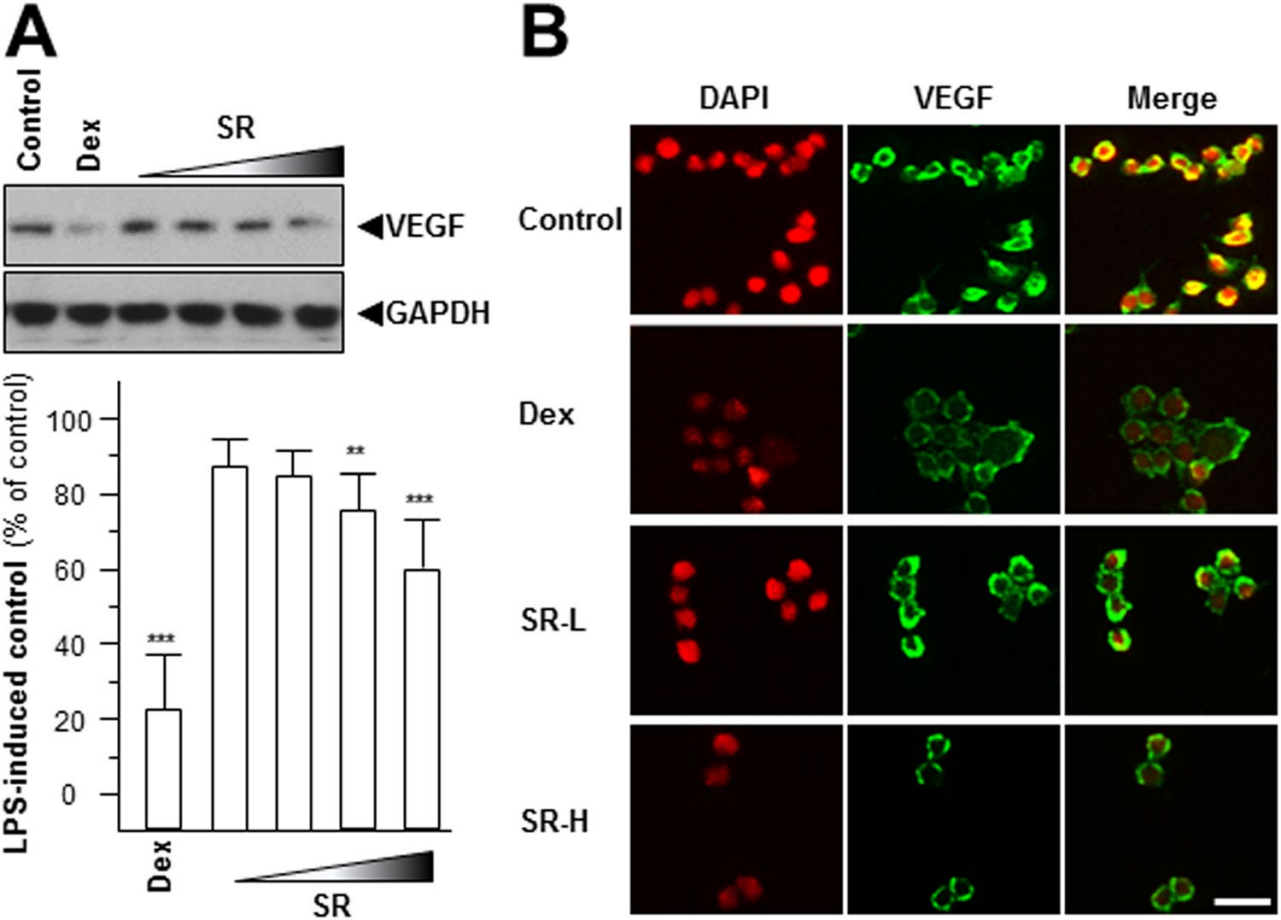
SR reduces the expression of angiogenic marker. Different dilutions of SR extracts (0.03, 0.1, 0.3, 1.0 mg/mL) were used onto LPS-stimulated RAW 264.7 cells for 48 hours. (**A**) The protein expression level of VEGF (~27 kDa) was detected by immunoblot analysis using specific antibodies (upper panel), and GAPDH (~38 kDa) served as an internal control. Here, dexamethasone (Dex; 10 μM) served as positive control. Values were shown as the percentage of LPS-induced maximum reading (lower panel), in Mean ± SEM, where *n* = 3. Statistically significant changes were classified as significant more significant (**) where *p* < 0.01 and highly significant (***) where *p* < 0.001 as compared with control group. (**B**) VEGF expression level was revealed by immunochemical staining. Bar = 10 µm.

The abnormal expressions of hypoxia-inducible factor-1 (HIF-1) and VEGF are the hallmark of hypoxia, inflammation, carcinogen invasion^12,20^. HIF-1 is a highly conserved transcriptional complex, and which is a heterodimer composed of α and β subunit. The expression of HIF-1α showed a closely relationship with hypoxia condition, instead of HIF-1β^20^. Here, the transcript levels of HIF-1α and HIF-1β were determined after application of SR herbal extract. The mRNA level of HIF-1α was declined by over 30% maximum, but not for HIF-1β (Fig. 5A). The protein level of HIF-1α was also significantly decreased: the maximal decline level was at ~60% (Fig. 5B). Moreover, the expression of VEGF was determined by western blot. The amount of VEGF, revealed by western blotting in LPS-stimulated RAW 264.7 cells, was robustly decreased after application of SR extract in a concentration-dependent manner **(**Fig. 6A**)**. The laser confocal method was also employed here to provide the detailed VEGF expression pattern. After high dose of SR (1.0 mg/mL, SR-H) treatment, the VEGF expression was markedly reduced, as compared to the LPS-treated control **(**Fig. 6B**)**. Dexamethasone served as a positive control, and which could mitigate VEGF expression level, significantly **(**Fig. 6A,B**)**. All of these results indicated that SR showed the possibilities of inhibiting inflammatory-induced angiogenesis.

### The SR-derived flavonoids suppress inflammation

**Figure 7:**
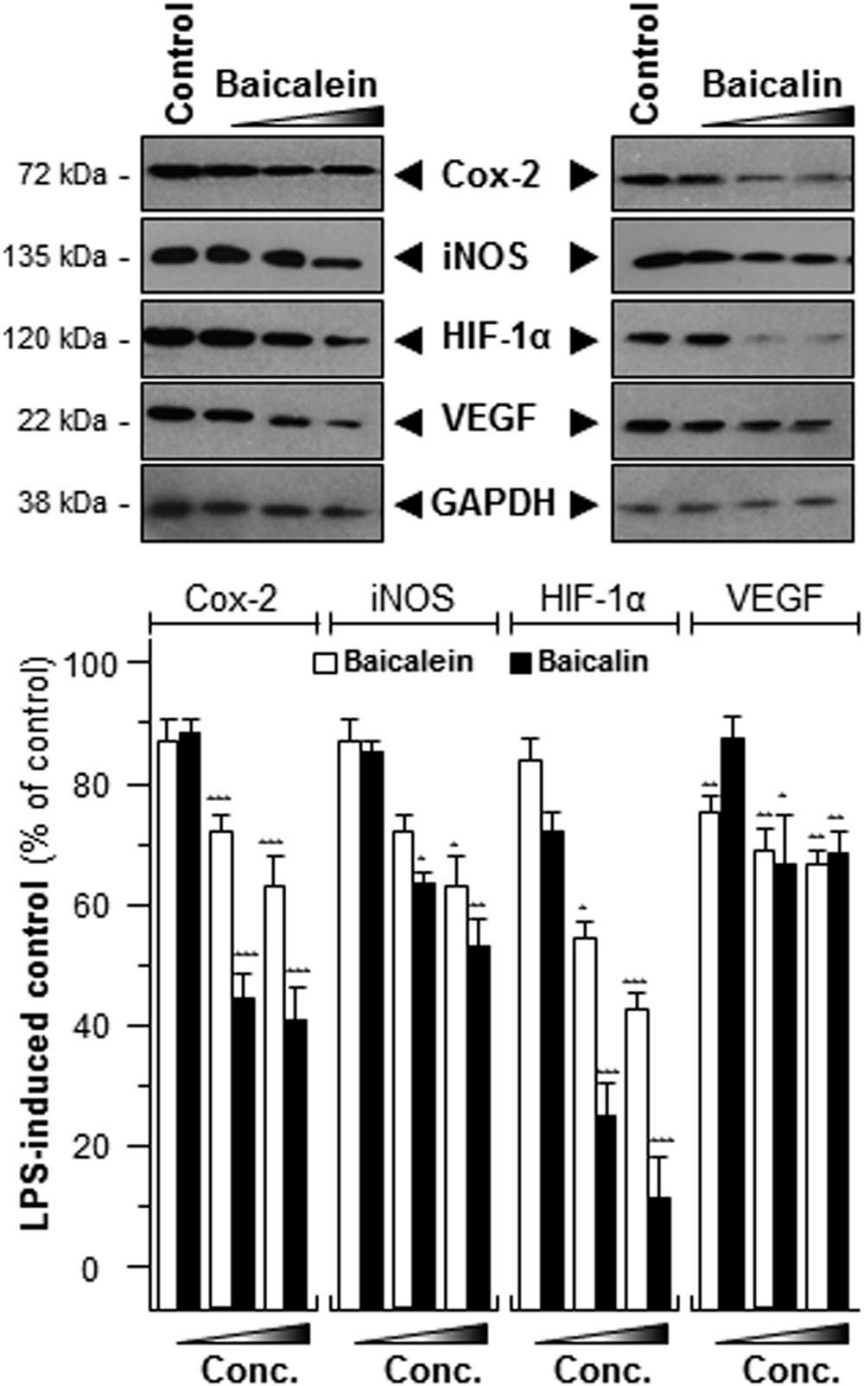
Baicalein and baicalin suppress the expressions of Cox-2, iNOS, HIF-1α and VEGF. The LPS-stimulated cells were treated with LPS (1 μg/mL) for 24 hours and then challenging with various concentrations of baicalin or baicalein (3, 10, 30 nM) for another 48 hours. The protein levels of Cox-2 (~72 kDa), iNOS (~130 kDa), HIF-1α (~90 kDa) and VEGF (~27 kDa) were detected by immunoblot analysis (upper panel), and GAPDH (~38 kDa) served as an internal control. Values were shown as the percentage of LPS-induced maximum reading (lower panel), in Mean ± SEM, where *n* = 3. Statistically significant changes were classified as significant (*) where *p* < 0.05 more significant (**) where *p* < 0.01 and highly significant (***) where *p* < 0.001 as compared with control group.

**Figure 8:**
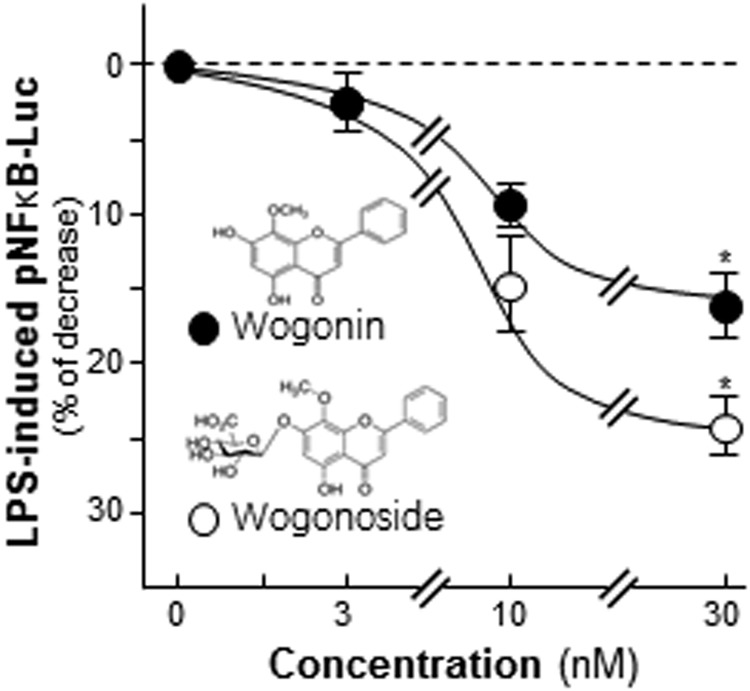
Wogonin and wogonoside inhibit the activity of pNFκB-Luc. A luciferase reporter contains 5 repeat NFκB response elements, named pNFκB-Luc, was applied here. Transfected cells were treated with LPS (1 μg/mL) for 24 hours and then application with wogonin or wogonoside at different concentrations for another 2 days. The cell lysates were subjected to luciferase assay. Chemical structures of wogonin and wogonoside were shown. Data were expressed as percentage of control and in Mean ± SEM, where *n* = 3. *p < 0.05 as compared to the control.

The anti-cancer functions of baicalin and baicalein, two major active components of SR, have been reported^21,22^. Inflammation plays an indispensable role for tumorigenesis^9,23^. Accordingly, the anti-inflammation properties of baicalin and baicalein were explored here. The results indicated that these two chemicals have potential anti-inflammatory functions by reducing the translational activities of Cox-2 and iNOS, at different levels **(**Fig. 7**)**. Higher concentrations of baicalin and baicalein showed stronger suppressive activities on the LPS-induced inflammation **(**Fig. 7**)**. Suppression in inflammatory-specific genes can be attributed to inhibition of angiogenesis activity in LPS-induced RAW 264.7 cells **(**Fig. 7**)**. In addition, other flavonoids in SR, e.g. wogonin, wogonoside, were also reported to mediate the inflammatory processes both *in vitro* and *in vivo*^24–27^. Therefore, we analyzed the anti-inflammation functions of these two flavonoidic compounds in cultured RAW 264.7 cells **(**Fig. 8**)**. A promoter construct having NFκB activating DNA elements upstream of a luciferase gene was used in transfecting RAW 264.7 cells. In the DNA transfected cells, wogonin or wogonoside could suppress the LPS-induced transcriptional activity of pNFκB-Luc in a dose-dependent manner. The maximal suppression levels were at ~17% for wogonin and ~25% for wogonoside (Fig. 8**)**. These data suggested that SR and its major flavonoidic compounds, e.g. baicalein, baicalin, wogonin and wogonoside, could modulate inflammatory-induced angiogenesis in cultured macrophage.

## Discussion

According to TCM theory, health preservation is to preserve individual body to maintain health, prevent from diseases and prolong life expectancy. About 1,892 types of TCMs and over 11,000 Chinese herbal medicine prescriptions were recorded in Bencao Gangmu by Li Shizhen (AD ~1596). Today’s practices of TCMs are the culmination of theoretical development and clinical investigation over thousands of years in China. Indeed, TCM practices are believed to be based on the cosmologic principles of Chinese philosophy viewing disease as an imbalance of the living system, and therefore TCM treatments aim to maintain a balance. It is argued that this regimen is more suitable for chronic disease prevention and treatment^28^. In contrast, western medicine is relying on a detailed classification of disease based on empirical investigations and treatments. Synthesized drugs, usually in the single-chemical form, have been successful in treatment of acute conditions: this strategy is trying to influence the entire system by perturbing the single action. Many diseases are multi-factorial. In the case of chronic diseases, i.e. inflammation, the patients are suggested to intake medicines for a long and indefinite period of time, which often results in triggering serious side effects. Therefore, there is an increasing interest in the pharmaceutical industry by utilizing the herbal medicine as the novel candidates for the regimen of chronic diseases, i.e. inflammation.

The immune system has several defense mechanisms with increasing specificity to against the entry of pathogens and to avoid diseases. Innate immune cells, e.g. macrophages, usually orchestrate the rapid immune response by secreting different kinds of cytokines^29^. These cytokines play vital roles in monitoring immune response under pathogen infections and inflammations^29^. Chronic inflammation is a long-lasting disease and finally could result in development of cancer, cardiovascular diseases, neurodegenerative diseases and respiratory diseases^30^. Studies have shown that the transcription factors NFκB and STAT3, regulating the immune-related gene expressions, are essentially active during angiogenesis. Furthermore, the activations of NFκB and STAT3 could result in cancer cell proliferation, survival, invasion and metastasis by reducing the sensitivity to chemotherapy^30,31^. In fact, inflammation and angiogenesis are two closely related processes, and both of them could be triggered by hypoxia^32^. The anti-inflammatory agents are able to mitigate hypoxia condition and thereafter to alleviate angiogenesis. Moreover, the anti-inflammatory medicines are now widely accepted for angiogenic treatment in cancer therapy^32^. Together with our current results, the anti-angiogenic effect of SR in inflammatory macrophages could be mediated by multiple mechanisms: (i) suppressing pro-inflammatory cytokine expression; (ii) alleviating hypoxia condition by decreasing HIF-1α; and (iii) reducing angiogenesis inducer VEGF. Thus, SR, or its active floavonoids, baicalein, baicalin, wogonin and wogonoside, could be a promising target in developing drugs for relieving the syndromes of chronic inflammatory-triggered angiogenesis.

SR is a well-known herb found within many multi-herb formulations, and the major aims of these formulae are to reduce inflammation and anti-cancer^33^. SR is a key herb found within Xiaochai Hutang, a herbal formula written by Zhang Zhongjing in AD ~200. The major functions of this herbal decoction were to enhance immune system and to clear the “fire”. After intake of Xiaochai Hutang for 5 years in hepatitis patients, the liver function was greatly improved by 78%, and in parallel the serum levels of liver enzymes were reduced^34^. The *in vitro* study reported that this herbal formula not only suppressed wild-type virus number but also lamivudine-resistant HBV mutant^35^. The mechanistic study has revealed that the treatment with Xiaochai Hutang decreases the DNA-binding activity of nuclear extract of HepA2 cells to a specific cis-element of the HBV core promoter^35^. In cultured macrophages, the combination of SR and Liriopis Tuber could inhibit the expressions of inflammatory protein and granulocyte colony-stimulating factor in a dose-dependent manner. In addition, this herbal combination suppressed colony-stimulating factor and tumor necrosis factor at a dose of 25 μg/mL^36^. In parallel, the water extract of SR was able to induce apoptosis and to change the ratio of Bax/Bcl in a series of cancer cells^37^. Similarly, SR is selectively toxic to lung cancer cell lines by enhancing the expressions of p53 and Bax^38^. Oral administration of SR water extract for 10 days significantly inhibited tumor size in mouse xenograft model^39^. After oral administration of SR for 5 days in rat model, the level of PGE2 in LPS-stimulated macrophages was reduced robustly, and the pharmacodynamic interaction was proposed to be via the Cox–2 pathway^40^. The major metabolites were reported to be baicalin and baicalein by utilizing HPLC coupled with electrochemical detector^41^. From the results, AUC 0–24 hour values were 1.66 ± 0.34 µM and 19.8 ± 3.9 µM for baicalin, and 0.853 ± 0.065 µM and 10.0 ± 3.1 µM for baicalein, respectively^41^. Furthermore, the pharmacokinetic parameters of baicalin and baicalein, after oral administration of SR, were calculated and analyzed by the pharmacokinetic program^42^.

The anti-cancer effects of SR have been suggested to be triggered by baicalin and baicalein. Baicalin suppressed the growth of lymphoma and myeloma cells by regulating transcriptional and translational levels of phospholipid scramblase 1, a regulator of cell cycle and differentiation-related genes^43^. The anti-cancer functions of baicalein are contributed for ROS scavenging ability, abolishing NFκB activity and affecting cell cycle genes^43,44^. More importantly, baicalin and baicalein are believed to be promising candidates for chemotherapy adjuvant by not inducing possible mutations, a major problem of conventional anti-cancer drugs^21,22^. In addition, wogonin and wogonoside inhibited cancer cell proliferation in a concentration-dependent manner in various cell models^37^. Moreover, wogonin was capable of inducing HL-60 cell death both by stimulating DNA fragmentation and up-regulating apoptosis marker expressions^45^. Besides the anti-inflammatory functions of wogonoside and wogonin, the anti-hepatitis B virus (HBV) pharmaceutical values of these compounds were also demonstrated^27,46^.

## Materials and Methods

### Raw material and HPLC condition

The raw material of SR (the dried root of *S*. *baicalensis*) was obtained from Hebei province, which was authenticated by Dr. Tina Dong, one of the authors. The voucher specimen of SR (# 02-09-06) was kept in Centre for Chinese Medicine of HKUST. The raw material of SR was weighed and boiled in water for 2 hours, twice: the volume was 8 times and 6 times, respectively. Baicalin, baicalein, wogonin and wogonoside were purchased from TLCM (HKUST, Hong Kong China). The purities of these chemicals were, confirmed by HPLC, higher than 98.0%. HPLC analysis was conducted on an Agilent 1200 series system (Agilent, Waldbronn, Germany), equipped with a degasser, a binary pump, an auto-sampler, and a thermo-stated column compartment. Chromatographic separations were carried out on a Phenomenex C18 column (particle size 5 μm, 4.60 mm × 250 mm) with 1% acetate acid in water (as solvent A): acetonitrile (as solvent B) in the mobile phase at a flow rate of 1.0 mL/min at room temperature. A linear gradient elution was applied from 0 to 58% of solvent A starting from 0 to 60 min. 10 μL samples were injected for HPLC analysis. Analytical- and HPLC-grade reagents were from Merck (Darmstadt, Germany).

### Cell culture

RAW 264.7 cells, from American Type Culture Collection (ATCC, Manassas, VA), were cultured in Dulbecco’s modified Eagles medium equipped with 100 U/mL penicillin, 100 µg/mL of streptomycin and 10% heat in-active fetal bovine serum, at 37 °C incubator. The herbal extracts were lyophilized and dissolved in water at 100 mg/mL as a stock concentration. LPS (1 μg/mL) was applied for 24 hours as mimicking inflammatory situation, and then different concentration of SR extracts, or other chemicals, were applied. Here, Dex (10 μM) served as anti-inflammatory function’s positive control. The culture medium and reagents were from Life Technologies (Carlsbad, CA)

### Western blot analysis

The expressions of Cox-2, iNOS, HIF-1α and VEGF were revealed by western blot. Cultures were seeded onto 6-well plate for 48 hours. High salt lysis buffer (1 M NaCl, 10 mM HEPES, pH 7.5, 1 mM EDTA, 0.5% Triton X-100) was utilized for collecting cells. Total protein of each sample was adjusted by 2X lysis buffer (0.125 M HCl, pH 6.8, 4% SDS, 20% glycerol, 2% 2-meracptoethanol and 0.02% bromophenol blue), and which was subjected to SDS-PAGE analysis. The membranes were incubated with different antibodies, i.e. anti-Cox-2 (~72 kDa), iNOS (~135 kDa), HIF-1α (~120 kDa) and VEGF (~22 kDa) (CST, Danvers, MA). The above mentioned antibodies were at 1: 2,000 dilutions in the 2.5% fat-free milk. GAPDH was employed for the internal control at 1: 5,000,000 dilutions dissolved in the 2.5% fat-free milk. Horseradish peroxidase (HRP)-conjugated anti-rabbit and anti-mouse secondary antibodies (Zymed, South San Francisco, CA) were employed here as secondary antibody at 1: 5,000 dilutions for 3 hours at room temperature.

### Laser confocal detection for VEGF expression

VEGF level was measured after the challenging of SR extract (SR-H: 1 mg/mL; SR-L: 0.03 mg/mL) for 48 hours. Four % methanol-free paraformaldehyde was utilized here for sample dehydration and fix. Primary and secondary antibodies were dissolved in PBS containing 2.5% fetal bovine serum and 0.1% Triton X-100 (Sigma). Primary antibody was in PBST at 1:500 dilutions, and secondary antibody was in 1:1,000 dilutions, respectively. Finally, 1: 5,000 dilutions of DAPI was added onto the cells. Olympus Fluoview FV1000 laser scanning confocal system (LSCM) (Olympus, Melville, NY) mounted on an inverted Olympus microscope, equipped with a 100X objective, was used for fluorometric measurement.

### NO production detection

Fluorimetric measurements of nitric oxide (NO) were performed by LSCM. Intracellular NO production was evaluated by NO indicator 4-amino-5-methylamino-20, 70-difluorofluorescein (DAF-FM DA, Life Technologies). DAF-FM DA is not capable of reacting with NO itself but with NO^+^ equivalents, i.e. nitric anhydride (N_2_O_3_). Cells were incubated for 30 min at 37 °C in normal physiological solution containing 1 µM DAF-FM DM after processed. The amount of NO was evaluated by measuring the fluorescence intensity excited at 495 nm and emitted at 515 nm.

### Nuclear protein extraction

The translocation activation of NFκB (~65 kDa) was determined by Qproteome Nuclear Protein Kit (Qiagen, UK). In brief, nuclear and cytosol extract were isolated according to the manufacturer’s instruction after drug treatment. Twenty-five microliters of 0.2 µg/μL nuclear-isolated extract was dissolved in lysis buffer and then performed SDS-PAGE analysis. Anti-histone-1 (~27 kDa) was served as a loading control. After transferring, the membranes were incubated with specified antibodies. The working condition of anti-NFκB was at 1:5,000 dilutions and anti-histone-1 at 1:5,000 dilution. All the antibodies were kept at 4 °C for overnight.

### PCR analysis and DNA gel

Total RNA was extracted from the drug treated-cells with RNAzol reagent (Life Technologies) according to manufacturer’s instructions. RNA samples with OD260/OD280 ratios higher than 2.0 were used. One μg of total RNA was used for the production of cDNA, using a PCR system. The oligonucleotide primer sequence was as follows: IL-1β: 5′-AAA TAC CTG TGG CCT TG-3′, 5′-TTA GGA AGA CAC GGA TTC-3′; IL-6: 5′-GGA GTA CCA TAG CTACCT GG-3′, 5′-CTA GGT TTG CCG AGT AGA TC-3′; TNF-α: 5′-AGT GAC AAG CCT GTA GCC-3′, 5′-AGG TTG ACT TTC TCC TGG-3′; HIF-1α: 5′-GCT TTA ACT TTG CTG GCC CCA GC-3′; 5′-GCA GGG TCA GCA CTA CTT CGA AG; HIF-1β: 5′-ACT GGC AAC ACA TCC ACT GAT GGC-3′, 5′-CTG AAG TGG AAA GCT GCT CAC G-3′; GAPDH: 5′-AAC GGA TTT GGC CGT ATT GG-3′, 5′-CTT CCC GTT CAG CTC TGG G-3′. The PCR products were then run on 1.5% (v/v) agarose gels, stained with ethidium bromide, and photographed. The expression levels were quantified via scanning with a gel documentation and analysis system (Image J Program, NIH, Bethesda, MD, USA).

### Transfection analysis

The DNA construct containing 5X NFκB response element, named as pNFκB-Luc, was utilized here to detect the transcriptional activities upon the drug treatment. Lipofectamine 3000 (Invitrogen) was as a transfection kit, the transfection efficiency was over 30%. Cells were lysed by luciferase buffer containing 0.2% Triton X-100, 1 mM dithiothreitol (DTT) and 100 mM potassium phosphate buffer (pH 7.8) for luciferase assay after treated with LPS for 24 hours, then challenging with herbal extracts or chemicals for another 48 hours.

### Statistical analysis and other assays

Protein levels were evaluated by Bradford’s method (Herculues, CA). Statistical tests have been done by using one-way analysis of variance. Data were expressed as Mean ± SEM, where *n* = 3–5. Statistically significant changes were classified as significant (*) where *p* < 0.05, more significant (**) where *p* < 0.01 and highly significant (***) where *p* < 0.001 as compared with control group.

## References

[1] Turner MD, Nedjai B, Hurst T, Pennington DJ (2014). Cytokines and chemokines: at the crossroads of cell signaling and inflammatory disease. Biochim Biophys Acta..

[2] Newton K, Dixit VM (2012). Signaling in innate immunity and inflammation. Cold Spring Harb Perspect Biol..

[3] Navab KD (2011). Chronic inflammatory disorders and accelerated atherosclerosis: chronic kidney disease. Curr Pharm Des..

[4] Nonnenmacher Y, Hiller K (2018). Biochemistry of proinflammatory macrophage activation. Cell Mol Life Sci..

[5] Coussens LM, Werb Z (2002). Inflammation and cancer. Nature..

[6] Landskron, G., Fuente, M. D., Thuwajit, P., Thuwajit, C. & Hermoso, M. A. Chronic inflammation and cytokines in the tumor microenvironment. *J Immuno Res*. 149185 (2014). 10.1155/2014/149185 PMC4036716 24901008

[7] Deng Z (2017). Grades evaluation of Scutellariae Radix slices based on quality constant. Zhongguo Zhong Yao Za Zhi..

[8] Wang J (2015). Metabolomic study of Chinese medicine Huang Qin decoction as an effective treatment for irinotecan-induced gastrointestinal toxicity. RSC Advances..

[9] Ono M (2008). Molecular links between tumor angiogenesis and inflammation: inflammatory stimuli of macrophages and cancer cells as targets for therapeutic strategy. Cancer Sci..

[10] Mor F, Quintana FJ, Cohen IR (2004). Angiogenesis-inflammation cross-talk: vascular endothelial growth factor is secreted by activated T cells and induces Th1 polarization. J Immunol..

[11] Miyagi Masayuki, Uchida Kentaro, Takano Shotaro, Fujimaki Hisako, Aikawa Jun, Sekiguchi Hiroyuki, Nagura Naoshige, Ohtori Seiji, Inoue Gen, Takaso Masashi (2018). Macrophage-derived inflammatory cytokines regulate growth factors and pain-related molecules in mice with intervertebral disc injury. Journal of Orthopaedic Research®.

[12] Harmey JH, Dimitriadis E, Kay E, Redmond HP, Bouchier-Hayes D (1998). Regulation of macrophage production of vascular endothelial growth factor (VEGF) by hypoxia and transforming growth factor beta-1. Ann Surg Oncol..

[13] Hamrah P, Chen L, Zhang Q, Dana R (2003). Novel expression of vascular endothelial endothelial growth factor (VEGFR)-3 and VEGF-C on corneal dendritic cells. Am J Pathol.

[14] Gong AG (2017). Polysaccharide of Danggui Buxue Tang, an ancient Chinese herbal decoction, induces expression of pro-inflammatory cytokines possibly via activation of nfκb signaling in cultured Raw 264.7 cells. Phytother Res..

[15] Gately S, Li WW (2004). Multiple roles of COX-2 in tumor angiogenesis: a target for antiangiogenic therapy. Semin Oncol..

[16] Howe LR, Subbaramaiah K, Brown AM, Dannenberg AJ (2001). Cyclooxygenase-2: a target for the prevention and treatment of breast cancer. Endocr Related Cancer..

[17] Lim JW, Kim H, Kim KH (2001). Nuclear factor-kappa B regulates cyclooxygenase-2 expression and cell proliferation in human gastric cancer cells. Lab Invest..

[18] Aharonov O, Maftzir G, Benezra D (1993). The role of cytokines in angiogenesis. Ocul Immunol Inflamm..

[19] Förstermann U, Sessa WC (2012). Nitric oxide synthases: regulation and function. Eur Heart J..

[20] Krock BL, Nicolas S, Simon MC (2011). Hypoxia-induced angiogenesis. Genes cancer..

[21] Gao C (2017). Antitumor effects of baicalin on ovarian cancer cells through induction of cell apoptosis and inhibition of cell migration *in vitro*. Mol Med Rep..

[22] Dou J (2018). Baicalein and baicalin inhibit colon cancer using two distinct fashions of apoptosis and senescence. Oncotarget..

[23] Qu X, Tang Y, Hua S (2018). Immunological approaches towards cancer and inflammation: a cross talk. Front Immunol..

[24] Li HD (2017). Wogonin attenuates inflammation by activating PPAR-γ in alcoholic liver disease. Int Immunopharmacol..

[25] Wang J, Li K, Li Y, Wang Y (2017). Mediating macrophage immunity with wogonin in mice with vascular inflammation. Mol Med Rep..

[26] Yang YZ, Tang YZ, Liu YH (2013). Wogonoside displays anti-inflammatory effects through modulating inflammatory mediator expression using RAW264.7 cells. J Ethnopharmacol..

[27] Li C, Lin G, Zuo Z (2011). Pharmacological effects and pharmacokinetics properties of Radix Scutellariae and its bioactive flavones. Biopharm Drug Dispos..

[28] Wang BE (2000). Treatment of chronic liver diseases with traditional Chinese medicine. J Gastroenterol Hepatol..

[29] Zhong TY (2016). Hemocyanins stimulate innate immunity by inducing different emprotal patterns of proinflammatory cytokine expression in macrophages. J Immunol..

[30] Kunnumakkara AB (2018). Chronic diseases, inflammation, and spices: how are they linked?. J Transl Med..

[31] Fan Y, Mao R, Yang J (2013). NF-κB and STAT3 signaling pathways collaboratively link inflammation to cancer. Protein Cell..

[32] Muz B, Puente P, Azab F, Azab AK (2015). 2015. The role of hypoxia in cancer progression, angiogenesis, metastasis, and resistance to therapy. Hypoxia..

[33] Li-Weber M (2009). New therapeutic aspects of flavones: the anticancer properties of *Scutellaria* and its main active constituents wogonin, baicalein and baicalin. Cancer Treat Rev..

[34] Yamamoto H, Miki S, Deguchi H (1994). Five year follow up study of Sho-saiko-to (Xiao-Chai-Hu-Tang) administration in patients with chronic hepatitis. J Nissei Hosp..

[35] Tseng YP, Wu YC, Leu YL, Yeh SF, Chou CK (2010). Scutellariae Radix suppresses hepatitis B virus production in human hepatoma cells. Front Biosci..

[36] So, M. H. & Choi, Y. K. Anti-inflammatory effect of combination of Scutellariae Radix and Liriopis Tuber water extract. *Evid Based Complement Alternat Med*, 203965 (2015). 10.1155/2015/203965 PMC4641954 26604969

[37] Ikemoto S (2000). Antitumor effects of Scutellariae Radix and its components baicalein, baicalin, and wogonin on bladder cancer cell lines. Urology..

[38] Gao J, Morgan WA, Sanchez-Medina A, Corcoran O (2011). The ethanol extract of *Scutellaria baicalensis* and the active compounds induce cell cycle arrest and apoptosis including upregulation of p53 and Bax in human lung cancer cells. Toxicol Appl Pharmacol..

[39] Xu WR (2014). The possibility of traditional Chinese medicine as maintenance therapy for advanced non-small cell lung cancer. Evid Based Complement Alternat Med..

[40] Fong SY, Wong YC, Xie C, Zuo Z (2015). Herb-drug interactions between Scutellariae Radix and mefenamic acid: Simultaneous investigation of pharmacokinetics, anti-inflammatory effect and gastric damage in rats. J Ethnopharmacol..

[41] Akao T, Sato K, He JX, Ma CM, Hattori M (2013). Baicalein 6-O-β-D-glucopyranuronoside is a main metabolite in the plasma after oral administration of baicalin, a flavone glucuronide of Scutellariae Radix, to rats. Biol Pharm Bull..

[42] Zhang L, Xing D, Wang W, Wang R, Du L (2006). Kinetic difference of baicalin in rat blood and cerebral nuclei after intravenous administration of Scutellariae Radix extract. J Ethnopharmacol..

[43] Kumagai T (2007). *Scutellaria Baicalensis*, a herbal medicine: anti-proliferative and apoptotic activity against acute lymphocytic leukemia, lymphoma and myeloma cell lines. Leuk Res..

[44] Lee WR (2002). Wogonin and fisetin induce apoptosis in human promyeloleukemic cells, accompanied by a decrease of reactive oxygen species, and activation of caspase 3 and Ca(2+)‐dependent endonuclease. Biochem Pharmacol..

[45] HU CHENGJUN, XU MAOZHONG, QIN RUJUAN, CHEN WEIFENG, XU XIN (2015). Wogonin induces apoptosis and endoplasmic reticulum stress in HL-60 leukemia cells through inhibition of the PI3K-AKT signaling pathway. Oncology Reports.

[46] Huang Ray-Ling, Chen Chien-Chih, Huang Huey-Lan, Chang Chung-Gwo, Chen Chieh-Fu, Chang Chungming, Hsieh Ming-Tsuen (2000). Anti-Hepatitis B Virus Effects of Wogonin Isolated from Scutellaria baicalensis. Planta Medica.

